# Evaluation of five liquid culture media for rapid detection of *Mycobacterium tuberculosis*

**DOI:** 10.3389/fcimb.2025.1655595

**Published:** 2025-11-04

**Authors:** Yihui Xie, Zhaoqin Zhu, Yuxin Shi, Zihui Zhao, Xueqin Qian, Yinzhong Shen

**Affiliations:** ^1^ Department of Laboratory Medicine, Shanghai Public Health Clinical Center, Fudan University, Shanghai, China; ^2^ Department of Radiology, Shanghai Public Health Clinical Center, Fudan University, Shanghai, China; ^3^ Shanghai Institute of Infectious Disease and Biosecurity, Fudan University, Shanghai, China; ^4^ Department of Infection and Immunity, Shanghai Public Health Clinical Center, Fudan University, Shanghai, China

**Keywords:** *Mycobacterium tuberculosis*, liquid culture medium, BACTEC MGIT 960 medium, rapid culture, carrot extract

## Abstract

**Objective:**

The BACTEC MGIT 960 system is currently the most widely used rapid culture system for *Mycobacterium tuberculosis*. However, its reagents have complex compositions, expensive, and rely on import. This study aimed to develop a self-formulated culture with simple composition, low cost, and performance comparable to or even better than that of the BACTEC MGIT 960 system.

**Methods:**

Five experimental media were formulated by supplementing Sauton medium with carrot extract, potato extract, oleic acid, or choline chloride. These were compared against commercial MGIT 960 medium using *M. tuberculosis H37Rv* suspensions (10^-^³-10^-6^ mg/mL, triplicate cultures). Time to detection (TTD) and growth index (GI) were monitored automatically using the BACTEC MGIT 960 system.

**Results:**

For medium-high inocula (10^-^³-10^-5^ mg/mL), carrot- and potato-supplemented Sauton media showed equivalent performance to MGIT 960 (1-day longer TTD, P>0.05; comparable GI values). At low inoculum (10^-6^ mg/mL), MGIT 960 failed to detect growth within 42 days, whereas supplemented Sauton medium achieved positivity within 30 days. The carrot extract medium performed best (mean TTD = 20.2 days), followed by oleic acid and oleic acid + choline chloride formulations.

**Conclusion:**

Carrot- and potato-supplemented Sauton medium demonstrated comparable performance to the MGIT 960 system for specimens with medium-to-high bacterial loads. Carrot extract, oleic acid, and choline chloride significantly enhanced the detection rate of low-inoculum, with carrot extract exhibiting the most pronounced growth-promoting effects. This study validates an accessible alternative to expensive imported media, particularly beneficial for resource-limited settings.

## 1 Introduction

Since Robert Koch’s discovery of *Mycobacterium tuberculosis* in 1882, tuberculosis (TB) has remained a critical global public health challenge. Despite remarkable advancements in mycobacterial culture techniques, antimicrobial development, and vaccine research over the past century, TB persists as a leading cause of mortality worldwide, ranking among the top 10 causes of death (World Health Organization, 2024). While molecular diagnostic tools like GeneXpert MTB/RIF have significantly improved diagnostic efficiency (Chakravorty et al., 2017), mycobacterial culture remains the irreplaceable gold standard for TB control, epidemiological surveillance, and precision medicine (Clinical and Laboratory Standards Institute, 2023).

*M. tuberculosis* is an obligate aerobe with an inherently slow growth rate due to its genetic determinant, exhibiting a doubling time of 15–20 hours. Conventional solid media require 2–5 weeks for visible colony formation, while liquid cultures still necessitate 10–15 days (Pfyffer, 2015; Vilchèze and Jacobs, 2019). Among rapid culture systems, the BACTEC MGIT 960 system (BD960; Becton Dickinson) has emerged as the most widely adopted global platform for combined culture, identification, and drug susceptibility testing, achieving a mean time to detection of 14.4 days (Dickinson, 2022).Although the BD960 system offers sensitive, rapid, and specimen-flexible detection, its reliance on expensive imported reagents severely limits accessibility in resource-limited settings. The 2023 WHO TB Diagnostic Guidelines highlight insufficient liquid culture availability as a key bottleneck in global TB control, particularly in low-resource regions, and explicitly call for alternative media with comparable performance at halved costs (World Health Organization, 2023a).

To address this unmet need, our study innovatively developed a modified Sauton medium supplemented with cost-effective growth-promoting components like plant extracts, systematically evaluating its potential to replace commercial reagents, with the aim of providing an efficient and economical tuberculosis diagnostic solution for primary healthcare institutions.

## 2 Materials and methods

### 2.1 Instruments and reagents

The following materials were used in this study:

Culture system: BACTEC MGIT 960 fully automated mycobacterial growth indicator tube system (BD Diagnostics), Culture media components: MGIT culture tubes, PANTA antibiotic mixture (BD), OADC enrichment supplement (oleic acid, albumin, dextrose, and catalase; BD);

Chemical reagents: Oleic acid (Sangon Biotech), Choline chloride (Sangon Biotech), Glycerol (Sangon Biotech), KH_2_PO_4_ (Sangon Biotech), MgSO_4_·7H_2_O (Aladdin), Citric acid (Sangon Biotech), Ammonium ferric citrate (Sangon Biotech), L-Asparagine (Merck), Human plasma (Jinshan District Blood Center), Plant-derived supplements: Freshly purchased potatoes and carrots;

Staining reagent: BASO™ TB staining kit (Zhuhai Beisuo Biotechnology);

### 2.2 Bacterial strain

The *Mycobacterium tuberculosis* H37Rv reference strain (laboratory stock culture) was sub-cultured on Löwenstein-Jensen (L-J) medium for 2–3 weeks prior to experiments.

### 2.3 Preparation of culture medium

#### 2.3.1 Preparation of Sauton medium

The composition and preparation method are detailed in **Table 1**.

**Table 1:** Formulation and preparation protocol of Sauton medium.

Component	Amount	Preparation method
Glycerol	60mL	1. Dissolve all components in 800 mL distilled water. 2. Adjust pH to 7.2 with 6mol/L NaOH. 3. Bring final volume to 1000 mL with distilled water. 4. Sterilize by autoclaving at 121 °C for 20 min. 5. Aliquot under sterile conditions. (*Final volume adjustment)
KH_2_PO_4_	0.5g	
MgSO_4_	0.5g	
Citric acid	2.0g	
Ammonium ferric citrate	0.05g	
L-Asparagine	4.0g	
Distilled water	970mL*	

#### 2.3.2 Preparation of carrot extract

Fresh carrots (20g) were diced and homogenized with 300mL of distilled water. The mixture was boiled for 20 minutes and then filtered through sterile gauze. A 100mL aliquot of the clarified filtrate was incorporated into Sauton medium, replacing an equivalent volume of distilled water.

#### 2.3.3 Preparation of potato extract

Fresh potatoes (20g) were diced and homogenized with 300mL of distilled water. The mixture was boiled for 20 minutes and then filtered through sterile gauze. A 100mL aliquot of the clarified filtrate was incorporated into Sauton medium, replacing an equivalent volume of distilled water.

#### 2.3.4 Preparation of potato-carrot extract

Fresh carrots (20g) and potatoes (20g) were diced and homogenized with 300mL of distilled water. The mixture was boiled for 20 minutes and then filtered through sterile gauze. A 100mL aliquot of the clarified filtrate was incorporated into Sauton medium, replacing an equivalent volume of distilled water.

#### 2.3.5 Oleic acid-supplemented medium

Oleic acid (5µg, purchased from Sangon Biotech) was added to 100mL of Sauton medium.

#### 2.3.6 Oleic acid-choline chloride-supplemented medium

A combination of 5µg oleic acid and 20mg choline chloride was supplemented into 100mL of Sauton medium.

**Figure 1:**
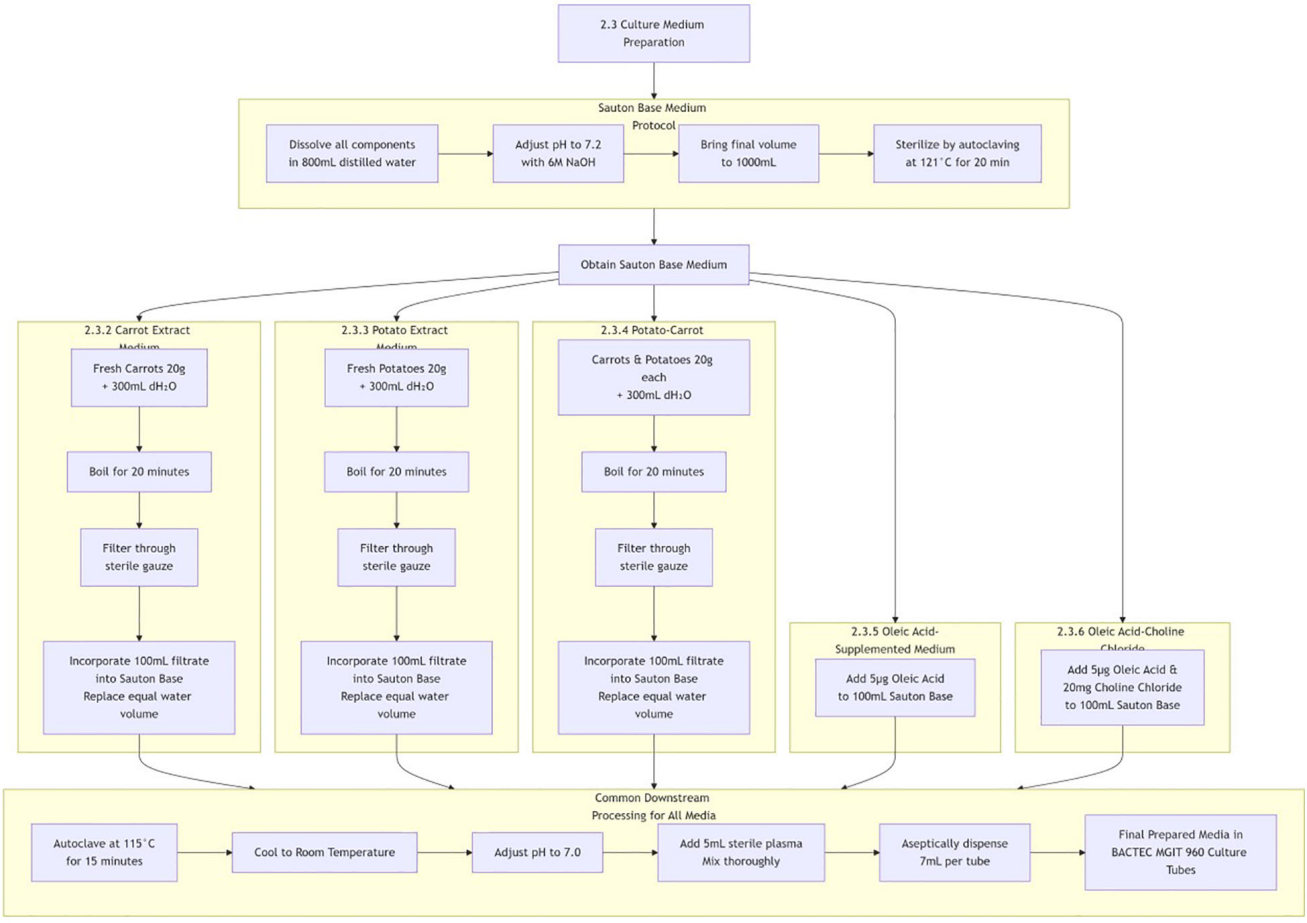
Preparation flowchart of five self-formulated media.

All media were autoclaved at 115°C for 15 min, cooled to room temperature, and adjusted to pH 7.0. Then 5mL of sterile plasma was added and mixed thoroughly. The prepared media were aseptically dispensed (7mL per tube) into sterile BACTEC MGIT 960 culture tubes. **Figure 1** illustrates the entire preparation process of five self- formulated media in the form of a flowchart.

### 2.4 Inoculation: preparation and inoculation of bacterial suspensions

Freshly sub-cultured H37Rv strains grown on Löwenstein-Jensen medium were harvested using 3mL of sterile distilled water and transferred into sterile glass grinding tubes. The suspensions were vigorously vortexed and allowed to settle for 15 min to achieve a turbidity equivalent to 1 McFarland standard (1 McFarland ≈ 1mg/mL). Serial 10-fold dilutions were prepared in distilled water to obtain bacterial suspensions ranging from 1×10^-^³ to 1×10^-6^mg/mL (10mL per dilution). Each dilution (240µL) was inoculated into triplicate tubes containing the five test media or BACTEC MGIT 960 culture broth. **Supplementary Figure 1** displays the culture media and the BD960 automated culture system used in this study.

### 2.5 Result monitoring

Inoculated tubes were loaded into the BACTEC MGIT 960 System for automated mycobacterial growth detection. The instrument continuously monitored fluorescence signals and flagged positive samples hourly. Upon positivity detection, tubes were removed, and the time-to-detection (TTD) and growth index (GI) were recorded. A 3mL aliquot from positive cultures was treated with 3mL of 5% sodium hypochlorite, followed by centrifugation. The sediment was smeared onto slides for Ziehl-Neelsen staining, and the presence of acid-fast bacilli (AFB) was confirmed by microscopic examination. Subsequently, a 100µL aliquot from AFB-positive cultures was applied to MPB64 immuno-chromatographic assay strips for identification of *Mycobacterium tuberculosis*. All AFB-positive samples yielded positive MPB64 test results. Cultures not flagged as positive within six weeks were considered negative.

### 2.6 Statistical analysis

Statistical analysis was performed using R software (version 4.4.0). The Wilcoxon rank-sum test (Mann-Whitney U test) was applied to compare time to detection (TTD) and growth index (GI) values between experimental and control groups, with a significance threshold of P< 0.05.

Growth Index (GI) Definition: The BACTEC MGIT 960 Mycobacterial Growth Indicator System quantifies mycobacterial growth intensity using GI, calculated as follows: Each MGIT tube contains an oxygen-sensitive fluorescent indicator at the bottom. As mycobacteria grow, they consume oxygen, increasing fluorescence intensity. The system monitors fluorescence changes in real-time and computes GI using the formula:


GI=k•(ΔF/Δt)

- ΔF = change in fluorescence intensity per unit time
- k = instrument calibration constant

## 3 Results

### 3.1 Comparative growth characteristics across culture media

As demonstrated in **Table 2**, the five tested media exhibited significant variations in their capacity to support the growth of *Mycobacterium tuberculosis* H37Rv. Notably, the Sauton + carrot + potato medium yielded nonspecific positivity signals within 3 days post-inoculation; subsequent acid-fast staining confirmed negative with no contamination observed, leading to its exclusion from further analysis. The remaining four media consistently supported mycobacterial growth.

**Table 2:** Growth characteristics of M. tuberculosis in different media.

Medium group	Inoculum (mg/mL)	TTD (days, mean ± SD)	GI (mean ± SD)	P-value (vs. Control)
BACTEC MGIT 960 (Control)	1×10^-^³	10.33 ± 0.29	2085 ± 125	—
	1×10^-4^	13.25 ± 0.38	137 ± 18	—
	1×10^-5^	17.83 ± 0.42	629 ± 45	—
	1×10^-6^	>42	—	—
Sauton + Carrot	1×10^-^³	11.46 ± 0.52	504 ± 32	0.08
	1×10^-4^	12.25 ± 0.41	148 ± 15	0.12
	1×10^-5^	18.29 ± 0.47	249 ± 22	0.15
	1×10^-6^	20.17 ± 0.85	115 ± 12	**<0.05**
Sauton + Potato	1×10^-^³	11.00 ± 0.45	1406 ± 98	0.10
	1×10^-4^	14.21 ± 0.39	177 ± 16	0.14
	1×10^-5^	18.92 ± 0.51	284 ± 25	0.18
	1×10^-6^	>42	—	—
Sauton + Oleic Acid	1×10^-^³	18.83 ± 1.24	256 ± 28	**<0.01**
	1×10^-4^	24.75 ± 1.18	1178 ± 105	**<0.01**
	1×10^-5^	26.50 ± 1.12	1374 ± 118	**<0.01**
	1×10^-6^	28.42 ± 1.13	118 ± 14	**<0.05**
Sauton + Oleic Acid + Choline Chloride	1×10^-^³	17.67 ± 1.05	1219 ± 102	**<0.01**
	1×10^-4^	22.42 ± 1.10	7450 ± 325	**<0.01**
	1×10^-5^	28.79 ± 1.26	330 ± 29	**<0.01**
	1×10^-6^	32.88 ± 1.26	924 ± 78	**<0.05**

Data are presented as mean ± standard deviation (x̄ ± SD).”>42” indicates no detection within the 42-day incubation period. P-value was determined using the Mann-Whitney U test; statistically significant differences (P< 0.05) are highlighted in bold.

### 3.2 Culture performance under moderate-to-high inoculum conditions

Under moderate-to-high inoculum conditions (10^-^³-10^-5^ mg/mL), both carrot- and potato-supplemented Sauton culture media (Media ① and ②, respectively) exhibited comparable culture efficiency to the commercial MGIT 960 medium, with a marginal 24-hour prolongation in mean time to detection (TTD) and statistically equivalent growth index (GI) values (P > 0.05).In marked contrast, oleic acid-containing formulations (Media ④ and ⑤) showed substantially extended TTDs: Sauton + oleic acid: 7–9 days delay versus control (P< 0.01) and Sauton + oleic acid + choline chloride: 6–8 days delay (P< 0.01).

### 3.3 Sensitivity analysis at low inoculum (10^-6^ mg/mL)

At ultralow bacterial loads, statistically significant differences were observed: while BACTEC MGIT 960 failed to detect growth within the 42-day incubation period, three experimental media successfully achieved detection (P< 0.05 for all): Sauton + carrot: TTD = 20.17 ± 0.85 days; Sauton + oleic acid: TTD = 28.42 ± 1.13 days; Sauton + oleic acid + choline chloride: TTD = 32.88 ± 1.26 days. GI analysis further revealed that Sauton + carrot exhibited significantly higher GI values than other formulations (P< 0.05), demonstrating superior enhancement of bacterial metabolic activity under low-inoculum conditions.

## 4 Discussion

The 2024 WHO Global Tuberculosis Report revealed a staggering global incidence of 10.8 million new TB cases in 2023, with China accounting for 741,000 cases-ranking third among the 30 high-burden countries (World Health Organization, 2024).While molecular diagnostic advances have significantly reduced TB detection time, mycobacterial culture remains indispensable for cases of negative molecular tests or smear-negative pulmonary TB. The widely-used BACTEC MGIT 960 system, despite its sensitivity and rapidity, faces significant limitations in resource-limited settings due to its dependence on expensive imported reagents and complex supply chain requirements. Recent studies by domestic researchers have explored plant-derived alternatives (e.g. potato, coconut water) to replace imported medium components (Huang et al., 2005; Gong et al., 2007; Ling-Fei and Yan-Hong, 2009), though systematic comparative studies remain scarce. This study innovatively combined traditional Sauton medium with various growth-promoting supplements and rigorously evaluated their potential as alternatives to commercial reagents through controlled experiments.

Sauton medium, a classical non-selective liquid medium containing essential nutrient such as aspartic acid and ferric ammonium citrate for Mycobacterium tuberculosis growth, has been widely used for mycobacterial isolation and identification. Potato and carrot, being cost-effective and readily available, provide carbohydrates, lipids, proteins, and trace elements (e.g. Ca, Mg, Zn, Cu) crucial for mycobacterial growth (Tan, 2000). In this study, modified Sauton-potato and Sauton-carrot media demonstrated comparable culture efficiency to the commercial BACTEC MGIT 960 medium at inoculum concentrations of 10^-^³–10^-5^ mg/mL, with only a 24-hour delay in time to detection (TTD) (P > 0.05). These findings align with previous reports by Kuang et al (Kuang et al., 1985; Kuang and Wang, 1990), who demonstrated that potato extract significantly shortened culture times compared to the Löwenstein-Jensen medium for various mycobacteria, including *Mycobacterium tuberculosis*, *Mycobacterium bovis*, and *Mycobacterium intracellulare*. Notably, Sauton-potato medium has shown efficacy in industrial applications like BCG vaccine production (Dang et al., 2007). Importantly, the Sauton-carrot medium detected mycobacteria at ultralow inoculum (10–^6^ mg/mL) with a TTD of 20.17 ± 0.85 days, while BACTEC MGIT 960 did not detect growth within the 42-day incubation period (P< 0.05). The underlying mechanism may involve the following aspects: (1) The antioxidant activity of β-carotene (1.99-2.1mg/100g in carrots) potentially reduces ROS-mediated bacterial damage (Sies and Stahl, 1995); (2) Zinc trace elements upregulate the expression of zinc uptake regulator (Zur) genes, thereby enhancing the low-nutrient adaptability of Mycobacterium tuberculosis (Dow et al., 2021); (3) Magnesium ions synergize with components in Sauton medium to activate the PhoPR two-component system, precisely regulating mycobacterial lipid metabolism and redox homeostasis (Walters et al., 2006; Garcia et al., 2018); (4) Carrot polysaccharides can be enzymatically degraded into utilizable carbon sources to support early-stage bacterial proliferation (Baughn and Rhee, 2014). This study is the first to demonstrate the growth-promoting effects of carrot extract on low-inoculum *M. tuberculosis*, with the mechanism likely originating from the synergistic effects between β-carotene and various trace elements. These findings provide novel research directions for in-depth exploration of mycobacterial nutritional metabolism regulation mechanisms. Notably, this discovery not only fully complies with the technical requirements for improving extra-pulmonary tuberculosis diagnostic sensitivity outlined in the WHO Tuberculosis Diagnostic Guidelines (World Health Organization, 2021), but also offers an innovative solution for developing cost-effective, high-efficiency mycobacterial culture systems.

The mycobacterial cell wall contains over 60% lipid by dry weight, highlighting the crucial role of lipid metabolism in bacterial growth (Kuang et al., 1995). This characteristic was first established by Dubos et al. in 1945, who showed that adding long-chain fatty acids to protein-free media supported rapid growth of *M. tuberculosis* and *Mycobacterium bovis* in liquid culture within three days (Dubos and Middlebrook, 1947). Oleic acid exhibits a concentration-dependent dual effect: it promotes growth at optimal levels (0.005–0.05 μg/mL) but inhibits growth at higher concentrations (Zheng and Gao, 1965). This finding aligns with the WHO core principle of “optimizing culture medium composition for resource-limited settings” (World Health Organization, 2023b). Furthermore, it provides experimental validation for the use of oleic acid within the concentration range of 0.005–0.05μg/mL. Regarding the mechanism of choline chloride, current research remains contentious. Finlayson et al. reported no growth-promoting effects (Finlayson, 1946), whereas Zheng et al. demonstrated a 35–68% increase in proliferation rates (P< 0.05) at 12.5–200μg/mL (Zheng, 1962). This study provides novel evidence supporting the latter: at an ultralow inoculum (10^-6^mg/mL), all modified media containing oleic acid and/or choline chloride successfully detected H37Rv, while the commercial BACTEC MGIT 960 system failed within the 42-day incubation period.

This study provides the first experimental evidence demonstrating the significant growth-promoting effect of carrot extract on paucibacillary *Mycobacterium tuberculosis* strains. Notably, the production costs of potato- and carrot-based media represent less than 1/40 of commercial BACTEC MGIT 960 reagents (**Supplementary Table 1**), while offering additional advantages in terms of raw material accessibility and simplified preparation protocols—characteristics particularly suited for implementation in resource-limited primary healthcare settings. Under low bacterial load conditions (10^-6^mg/mL), the modified media exhibited significantly improved detection sensitivity, offering a promising solution for the diagnosis of extra-pulmonary and smear-negative tuberculosis. Furthermore, this study confirms the synergistic interactions between plant-derived components and lipid/choline supplements, establishing a novel conceptual framework for developing cost-effective diagnostic strategies for tuberculosis.

The global TB control situation continues to present significant concerns. As reported by the World Health Organization, an estimated 10.8 million individuals developed TB in 2023, among which nearly 3 million failed to receive timely diagnosis or treatment (World Health Organization, 2024). This diagnostic gap is particularly acute in resource-limited settings and primary healthcare facilities, where reliance on insensitive smear microscopy or slow solid culture persists, compounded by constraints in funding, staffing, and access to sophisticated equipment. While this study utilized the BD960 system as a reference standard for media assessment, we recognize its impracticality for widespread deployment in peripheral laboratories. Notably, our modified Potato-Sauton and Carrot-Sauton media show promising potential as alternatives to commercial culture media in terms of growth performance. These formulations benefit from readily available raw materials and straightforward preparation protocols. Furthermore, the incorporation of water-soluble tetrazolium salts as redox indicators enables visual detection of mycobacterial growth, offering a feasible approach for localized production of liquid culture media in basic-level laboratories. Therefore, the simple and accessible cultivation system we have developed demonstrates high feasibility and accessibility, even in resource-limited settings.

In the future, building upon our current work, we will advance the optimization and practical validation of our culture system across diverse settings. The subsequent work will focus on three key directions: Expand the spectrum of validated bacterial strains by incorporating representative fast- and slow-growing nontuberculous mycobacteria to systematically evaluate media performance across diverse growth characteristics; Advance to clinical sample validation through optimized antibiotic combinations and comprehensive assessment of various specimen types with different bacterial loads, including smear-negative samples; Enhance the supporting culture system framework by standardizing specimen processing methodologies, culture conditions, and interpretation criteria to improve practical utility and reliability. Through systematic translation from basic research to clinical implementation, we strive to establish an accessible, rapid, and economically viable mycobacterial culture system that can provide practical technical support for advancing global TB diagnostic capabilities and strengthening TB control initiatives.

## Data Availability

The original contributions presented in the study are included in the article/**Supplementary Material**. Further inquiries can be directed to the corresponding authors.
