# Supplementary figures and images for: Evaluation of five liquid culture media for rapid detection of Mycobacterium tuberculosis
Source: Front Cell Infect Microbiol. 2025 Nov 4;15:1655595. doi: 10.3389/fcimb.2025.1655595 (PMC12623306; doi:10.3389/fcimb.2025.1655595)

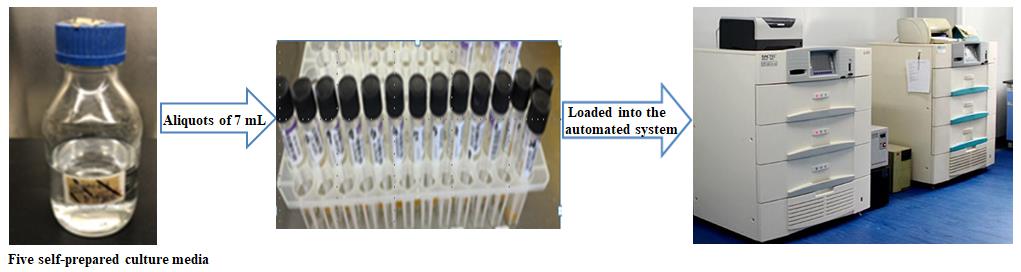

Supplement: Supplementary file 1 [file Image1.jpeg]
