# Supplementary material for: Evaluation of five liquid culture media for rapid detection of Mycobacterium tuberculosis
Source: Front Cell Infect Microbiol. 2025 Nov 4;15:1655595. doi: 10.3389/fcimb.2025.1655595 (PMC12623306; doi:10.3389/fcimb.2025.1655595)
Supplement: Supplementary file 2 [file Table1.docx]

| Component | Unit | Oleic Acid-Choline Chloride Medium | Oleic Acid Supplemented Medium | Potato Extract Medium | Carrot Extract Medium | Potato-Carrot Extract Medium |
| --- | --- | --- | --- | --- | --- | --- |
| **Base Components** | | | | | | |
| Glycerol | $/100ml | 0.162 | 0.162 | 0.162 | 0.162 | 0.162 |
| KH₂PO₄ | $/100ml | 0.0009 | 0.0009 | 0.0009 | 0.0009 | 0.0009 |
| MgSO₄ | $/100ml | 0.0127 | 0.0127 | 0.0127 | 0.0127 | 0.0127 |
| Citric Acid | $/100ml | 0.0143 | 0.0143 | 0.0143 | 0.0143 | 0.0143 |
| Ammonium ferric citrate | $/100ml | 0.0002 | 0.0002 | 0.0002 | 0.0002 | 0.0002 |
| L-Asparagine | $/100ml | 0.327 | 0.327 | 0.327 | 0.327 | 0.327 |
| **Special Supplements** | | | | | | |
| Oleic Acid (5μg) | $/100ml | <$0.000001 | <$0.000001 | - | - | - |
| Choline Chloride (20mg) | $/100ml | 0.0018 | - | - | - | - |
| Potato (20g) | $/100ml | - | - | 0.0165 | - | 0.0165 |
| Carrot (20g) | $/100ml | - | - | - | 0.0138 | 0.0138 |
| **Other** | | | | | | |
| Plasma (5ml) | $/100ml | 0.993 | 0.993 | 0.993 | 0.993 | 0.993 |
| **Total Cost** | **$/100ml** | **$1.512** | **$1.510** | **$1.541** | **$1.539** | **$1.555** |
| **Cost Per Tube** | **$/7ml** | **$0.106** | **$0.106** | **$0.108** | **$0.108** | **$0.109** |
| **BD960** | **$/7ml** | **$4.34** | | | | |
